# NGCN: Drug‐target interaction prediction by integrating information and feature learning from heterogeneous network

**DOI:** 10.1111/jcmm.18224

**Published:** 2024-03-20

**Authors:** Junyue Cao, Qingfeng Chen, Junlai Qiu, Yiming Wang, Wei Lan, Xiaojing Du, Kai Tan

**Affiliations:** ^1^ College of Life Science and Technology Guangxi University Nanning China; ^2^ School of Computer, Electronics and Information Guangxi University Nanning China

**Keywords:** drug‐target interaction, graph neural network, heterogeneous network, topology information

## Abstract

Drug‐target interaction (DTI) prediction is essential for new drug design and development. Constructing heterogeneous network based on diverse information about drugs, proteins and diseases provides new opportunities for DTI prediction. However, the inherent complexity, high dimensionality and noise of such a network prevent us from taking full advantage of these network characteristics. This article proposes a novel method, NGCN, to predict drug‐target interactions from an integrated heterogeneous network, from which to extract relevant biological properties and association information while maintaining the topology information. It focuses on learning the topology representation of drugs and targets to improve the performance of DTI prediction. Unlike traditional methods, it focuses on learning the low‐dimensional topology representation of drugs and targets via graph‐based convolutional neural network. NGCN achieves substantial performance improvements over other state‐of‐the‐art methods, such as a nearly 1.0% increase in AUPR value. Moreover, we verify the robustness of NGCN through benchmark tests, and the experimental results demonstrate it is an extensible framework capable of combining heterogeneous information for DTI prediction.

## 1 INTRODUCTION

The design and development of new drugs are a long process due to their high risk, long cycle and large investment. Also, the side effects of drugs on unexpected diseases and drug interactions have been shown to be potential risks to human health. Traditional biological experiments are effective in finding drug‐target interactions, whereas they are usually time‐consuming and costly.^1^, ^2^ Thus, computation approaches for detecting drug‐target interactions have recently become one of the most important parts of pharmacology development. With the growth of various drugs, targets and their interaction data, the computation‐based methods not only make predicting drug‐target interactions more economical and effective but also enhance the experiment reliability since they assist in explaining the mechanism of drug actions and their potential target activities.

Current prediction approaches for drug screening are mainly based on molecular docking,^3^ ligand similarity^4^ and machine learning.^5^

- The approach using molecular docking requires a known 3D structure of proteins, whereas the complex structures of known protein ligands are scarce and generally unavailable.
- The approach by ligand similarity employs the knowledge of known ligand interactions to make predictions. Nevertheless, if the target has insufficient ligands, the results may be poor.
- Machine learning is the most popular and effective approach at present, which can fully explore the relevant characteristics of drugs and the potential drug‐target interactions.

In recent years, many machine learning‐based methods have been proposed to predict potential DTIs. They mainly consist of the kernel method, matrix decomposition and multi‐source information integration.

According to chemical and genomic information, Yamanishi et al.^6^ used nuclear regression for DTI prediction and constructed a BLM model using bipartite graphs. Van Laarhoven et al.^7^ defined a gaussian interactive section core depending on the topological characteristics of the adjacency matrix and then used the kernel least squares (KRLS) algorithm to predict DTIs. Pahikkala et al.^8^ also employed the Kronecker regularized least squares (KRLS) algorithm, but they utilised the drug characterization based on 2D compound similarity and the Smith‐Waterman similarity characterization of the target. The kernel‐based methods only employ simple linear combinations, relying on several individual kernels to generate the final kernel matrix. This may be inappropriate if the linearity between the kernels is not obvious.

Matrix factorization is also widely used for DTI prediction. The dual‐nucleated Bayesian matrix decomposition (KBMF2K) proposed by Gonen et al.^9^ maps target proteins and drug compounds into the subspace of Bayesian by estimating the interaction network and using similarity in the subspace. Hao et al.^10^ established a drug‐target prediction model called DNILMF based on logical matrix decomposition. This model constructs two new kernel matrices, performs nonlinear diffusion between these two matrices and the two original similarity matrices, and predicts drug‐target interactions by gathering neighbour information. Ding et al.^11^ proposed a multiple kernel‐based triple collaborative matrix factorization (MK‐TCMF) method to predict DTIs. Multi‐kernel learning (MKL) algorithm can regulate the weight of each kernel matrix according to the prediction error. The aforementioned methods utilise direct drug‐target associations. This is challenging because the known information about the interaction is often incomplete.

With the rapid development of bioinformatics, various drugs, proteins, genes and other types of data have also been adopted for DTI prediction. Wan et al.^12^ constructed a large integrated network by combining data from multiple heterogeneous networks, captured the topological characteristics of the integrated network by using neighbourhood aggregation technology^13^ and reconstructed the topological representation of all relational matrices. Yu et al.^14^ developed an ensemble model (KenDTI) based on both biochemical characteristics of drugs via network integration and molecular sequences via word embedding to predict DTIs. Shao et al.^15^ regarded DTI prediction as a link prediction problem and proposed an end‐to‐end model based on heterogeneous graphs with attention mechanisms (DTI‐HETA). Fu et al.^16^ proposed a multi‐view graph convolutional network (MVGCN) framework for link prediction in biological networks by combining the similarity network to build a multi‐view heterogeneous network and obtain node attributes. In addition, a Neighbourhood Information Aggregation (NIA) layer was designed for inter‐ and intra‐domain information updating. Ren et al.^17^ integrated a large number of unlabeled drug molecular map information and target information and designed a pre‐training framework, MGP‐DR(molecular graph pretraining for drug representation), for drug pair representation learning. The model used a self‐supervised learning strategy to mine contextual information within and between drug molecules to predict drug–drug interactions and drug combinations. The graph convolutional neural network was utilised to obtain the embedded representation of the drugs and targets. The performance of network prediction tasks using graph convolution technology for large‐scale graph data has been significantly improved^18^ owing to the application of graph neural networks.^19^ In multi‐source data processing, it is usually easy to concatenate the features of different data sources. Therefore, how to make full use of the contributions of data from varied sources to efficiently fuse the DTI prediction is the key to improve the DTI prediction accuracy.

Motivated by the recent success of deep learning techniques in learning powerful representations from complex data,^20^, ^21^, ^22^, ^23^ Zhang et al.^24^ introduced related datasets for DTI prediction. Excluding the previously mentioned self‐supervised learning framework, MGPDR, introduced by Ren et al.,^17^ Chu et al.^25^ proposed the model, HGRL‐DTA, which was a novel approach for learning drug‐target binding affinity prediction through hierarchical graph representation. By incorporating both global affinity relationships and local chemical structures of drugs/target molecules, and utilising message broadcasting strategies, the model can synergistically integrate hierarchical information. The heterogeneous graph automatic meta‐path learning‐based DTI prediction method (HampDTI), proposed by Wang et al.,^26^ employed a node‐type specific graph convolutional network (NSGCN) to learn the embedding of drugs and targets using meta‐paths learned from a heterogeneous graph. The embedding from multiple meta‐path graphs has been combined to predict new DTIs.

The advantage of a deep learning method is its ability to identify hidden interactions between drugs and targets. However, they still have room for improvement in the following two aspects: (1) DTI prediction is to discover new DTIs. How to select truly interaction‐free drug‐target pairs is a thorny issue; (2) the fact that deep learning methods perform well on test datasets does not mean that they can also achieve good performance on discovering real drug.

This paper proposes a novel NGCN to predict DTIs. It can integrate various information from heterogeneous data sources, extract drug and target information from heterogeneous networks and reduce the feature information of drug or target to a low‐dimensional feature representation. Based on these low‐dimensional feature vectors, the spectral graph‐based convolutional neural (GCN) network is further applied to learn the drug or target features and avoid inaccuracy caused by the noise and incompleteness of large‐scale biological data. We compare NGCN with other methods to demonstrate its effectiveness and gradually increase the number of networks to prove the integration capability of NGCN. The results demonstrate that NGCN is promising for drug‐target interaction prediction.

## 2 PRELIMINARIES

Drug‐target interaction prediction of network syncretic aims to conduct prediction tasks by jointly utilising different views to exploit the complementarity.

Recently, there have been significant efforts towards integrating heterogeneous information from multiple networks. They can be roughly divided into two types of processes:

- Gather multiple networks to build a large integrated network and extract information for prediction.
- Extract feature information from each network and then fuse them for similarity or correlation prediction.

It is difficult to distinguish the discrepancies between different networks while constructing large integrated networks. And if the number of integrated networks is too large, computations on such a network will become challenging due to the increasing network complexity.

Extracting information from each network and making fusion predictions are the primary ways for drug‐target interaction prediction. The process is mainly composed of three steps: (1) extracting drug or protein information from each network; (2) feature fusion and dimensionality reduction; and (3) correlation prediction or drug relocation prediction based on extracted feature information.

Information extraction on a single network is the key step in network fusion. Common feature extraction consists of matrix decomposition and random walk with restart (RWR). The former usually decomposes the incidence matrix into two eigenvectors and minimises the loss of vector reconstruction. However, this strategy might lead to information loss and fail to capture the global characteristics of the incidence matrix.

As for RWR, a pre‐defined restart probability is introduced into the random walk with restart to identify the direct or indirect relationship between nodes of network. Suppose A and D are adjacency matrix and diagonal matrix, respectively. $D_{i,i}=\sum_{j=1}^{n}A_{i,j}$, the one‐step probability transition matrix $\hat{A}$ can be yielded by normalising the adjacency matrix.

(1) $$\hat{A}=D^{-1}A$$

Next, we introduce a t‐step RWR vector $r^{t}$, and $r_{i}^{t}$ means the probability of visiting node i after t step transitions. Let $r_{i}^{0}$ be the n‐dimensional initial one‐hot vector. A RWR process is defined as:

(2) $$r_{i}^{t+1}=\left(1-p\right)r_{i}^{t}\hat{A}+p*r_{i}^{0}$$

where p represents the probability of restart, and its value controls both global and local structural characteristics of the network. By iteratively executing the above process, we can get the diffusion state $r_{i}$ of the node, which is a high‐level representation of the structural characteristics in the network. Given two nodes in a network, if they share similar diffusion states, it means these two nodes have similar neighbourhood characteristics in the network.^27^

## 3 METHOD

The diffusion state is inaccurate, partially because the network data set in the experiment is noisy and incomplete. Luo et al.^27^ improved the diffusion component analysis method (DCA)^28^ and proposed the clusDCA for dimension reduction in the form of effective matrix decomposition. It is combined in our proposed model, NGCN, herein.

**FIGURE 1:**
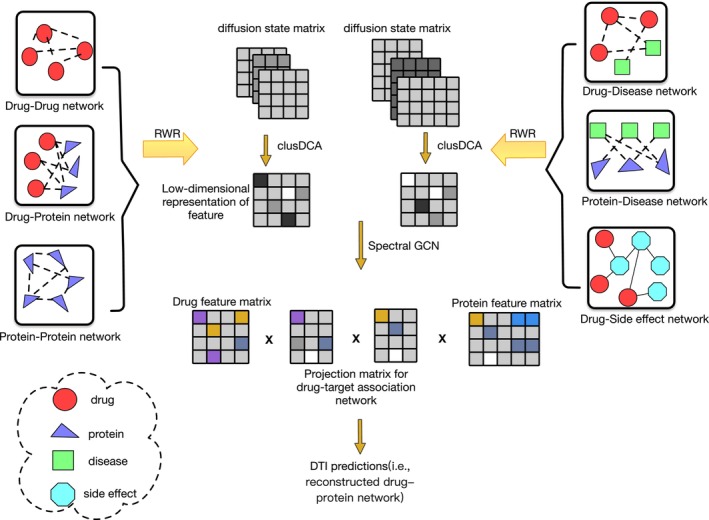
Workflow of NGCN. NGCN uses drug‐protein association network, protein–protein association network, drug–drug interaction network, drug‐disease network, protein‐disease association network and drug‐side effect network. We first obtain the diffusion state matrix (i.e. on each network to obtain a distribution of each drug or protein node, which captures its topological relations to all other nodes in the heterogeneous network) of each network through the RWR algorithm. The improved clusDCA algorithm is then used to calculate the low‐dimensional representation of the nodes. We add spectral GCN to update the node feature before reconstructing the drug‐target matrix. NGCN effectively learns topology‐preserving node features that are useful for predicting drug‐target interactions by enforcing the reconstruction of the original individual networks. Finally, the updated node properties are considered to reconstruct the drug‐target matrix.

The NGCN first conducts the RWR process on each drug or protein within each similar network to acquire the distribution of each drug or protein node, termed as the diffusion state. The diffusion state captures its topological relationship with all other nodes in the heterogeneous network. Subsequently, the improved clusDCA algorithm is employed to compute the low‐dimensional representation of the nodes. Leveraging the learned low‐dimensional features of drugs and proteins (where each row in the low‐dimensional drug features represents a feature vector of a drug and each column in the low‐dimensional protein features is a feature vector of a protein), NGCN executes spectral graph convolution to further refine the features of drugs and proteins. Finally, the drug‐target matrix is reconstructed to identify unknown drug‐target interactions. Details of the NGCN model are depicted in Figure 1.

### 3.1 Diffusion state of nodes by RWR

Our network data consists of homogeneous interaction networks, such as PPI network, and heterogeneous interaction networks, such as protein‐disease association networks. For the input homogeneous interaction networks (e.g. drug–drug interaction networks), we compute the “diffusion state” of each drug or target by directly running the RWR algorithm on each of these networks. As for heterogeneous interaction networks, we need to build similarity networks (e.g. to build protein–protein similarity network through protein‐disease association networks), perform the RWR on the derived similarity networks and then run the RWR process on these similarity networks to obtain the diffusion states of drugs or proteins. Overall, we construct similarity networks for drugs, based on (i) drug–drug interactions, (ii) drug‐disease associations and (iii) drug‐side‐effect associations. In the similar way, we construct similarity networks for proteins, based on (i) protein–protein interactions and (ii) protein‐disease associations.

Further, we can use the Jaccard similarity coefficient to calculate similarity between drugs, which is based on common neighbours and the union of sets of all neighbours of the two drugs. Given two nodes i and j, their similarity within a heterogeneous network is defined as:

(3) $$\mathrm{Sim}\left(i,j\right)=\frac{\left|\mathrm{Nod}e_{i}\cap \mathrm{Nod}e_{j}\right|}{\left|\mathrm{Nod}e_{i}\cup \mathrm{Nod}e_{j}\right|}$$

Then the diffusion state of each network can be obtained by running the RWR process on each similarity network, as described in Equ 2.

### 3.2 Performing feature reduction and feature extraction

Owing to the data quality and dimensionality issues, the diffusion state of drugs and targets produced by RWR may be error‐prone. In particularly, in case of the integration of multiple networks, it is often inconvenient to implement topological features directly by using the high dimensionality of the diffusion state. To address these problems and obtain important topological feature information about nodes from the diffusion state, we adopt a new diffusion component analysis method (clusDCA^29^) to perform feature reduction on diffusion state feature. Given node i, we model the probability assigned to node *j* in the diffusion state of node *i* as follows:

(4) $${\hat{s}}_{\mathrm{ij}}=\frac{\exp\left\{x_{i}^{T}w_{j}\right\}}{\sum_{j^{\prime }}\exp\left\{x_{i}^{T}w_{j^{\prime }}\right\}}$$

In order to reduce feature dimension more quickly and conveniently, clusDCA achieves rapid decomposition of the diffusion state via matrix decomposition. By modifying the formula, we have:

(5) $$\log{\hat{s}}_{\mathrm{ij}}=x_{i}^{T}w_{j}-\log\sum_{j^{\prime }}\exp\left(x_{i}^{T}w_{j^{\prime }}\right)$$

where $\forall i,x_{i}\in R^{d},w_{i}\in R^{d}$, *d* ≪ *n*. In this case, $w_{i}^{T}x_{j}$ is a low‐dimensional approximation, and the next term $\log\sum_{j^{\prime }}\exp\left(w_{i}^{T}x_{j^{\prime }}\right)$ represents a normalised factor. In our model, we remove this normalised factor $\log\sum_{j^{\prime }}\exp\left(w_{i}^{T}x_{j^{\prime }}\right)$ to eliminate the constraint that the sum of items in ${\hat{s}}_{i}$ is equal to 1, that is

(6) $$\log{\hat{s}}_{i}=x_{i}^{T}w_{i}$$

where $x_{i}$ and $w_{i}$ describe the topology of the network, $x_{i}$ represents the node feature, and $w_{i}$ can be regarded as the context characteristics of node i. The clusDCA takes a set of observed diffusion states $S=\left\{s_{1},\ldots ,s_{n}\right\}$ as input, and uses the sum of squared errors as the objective function:

(7) $$\min C\left(s,\hat{s}\right)=\sum_{i=1}^{n}\sum_{j=1}^{n}{\left(x_{i}^{T}w_{j}-\log{\hat{s}}_{\mathrm{ij}}\right)}^{2}$$

To optimise the objective function, we use singular value decomposition (SVD) in this process. Let *L* represent the logarithmic diffusion state matrix of the network. We define the SVD of the matrix *L* as follows:

(8) $$L=\mathrm{U\sum}V^{T}$$

where $U,\sum ,V\in R^{n\times n}$. Let the low‐dimensional feature matrix be $X=\left\{x_{1},\ldots ,x_{n}\right\}$. In terms of SVD, we calculate X as follows:

(9) $$X=U_{d}\sum_{d}^{0.5}$$

where $U_{d}$ represents the first *d* singular vectors and $\sum_{d}^{0.5}$ is the 0.5 power of the first singular values.

To integrate heterogeneous network data, DCA of the above single network needs to be extended to a multi‐network case. More specifically, let $L=\left\{L^{1},\ldots ,L^{K}\right\}$ denote the set of logarithmic diffusion state matrices obtained through the diffusion states $R_{c}=\left\{S^{1},\ldots ,S^{K}\right\}$ of *K* input networks. Then, the following objective function needs to be optimised:

(10) $$\min C\left(R_{c},{\hat{R}}_{c}\right)=\sum_{i=1}^{n}\sum_{j=1}^{n}\sum_{r=1}^{K}{\left(x_{i}^{T}w_{j}^{r}-\log{\hat{s}}_{\mathrm{ij}}^{r}\right)}^{2}$$

where $w_{j}^{r}$ represents the network‐specific feature of each node i in the network r, and the node feature $x_{i}$ is shared among all *K* networks. The above objective function can also be optimized by SVD.

### 3.3 Updating feature information

Although we have obtained the low‐dimensional representation of drug or target nodes, the node features need to be further updated due to the noisy and uncertain biological information. Here, we use the spectral graph‐based convolutional neural network for updating features.

Given the node feature $X^{\left(u\right)},u\in \left\{\text{drug},\text{protein}\right\}$, we update the features from each $X^{\left(u\right)}$ through spectral graph convolution to obtain a new representation of $X^{\left(u\right)}$. For the similarity network of $u\in \left\{\text{drug},\text{protein}\right\}$, we specify ${\tilde{A}}^{\left(u\right)}=A^{\left(u\right)}+I_{N}$ and diagonal matrix ${\tilde{D}}^{\left(u\right)}$ where ${\tilde{D}}_{\mathrm{ii}}^{\left(u\right)}=\sum_{j}{\tilde{A}}_{\mathrm{ij}}^{\left(u\right)}$. We then apply spectral convolution to obtain a new representation of nodes feature $H^{\left(u\right)}$:

(11) $$\begin{array}{rr} H_{u} & =f\left(X^{\left(u\right)},A^{\left(u\right)};W^{\left(u\right)}\right) \end{array}$$

(12) $$=\sigma \left({\hat{A}}^{\left(u\right)}X^{\left(u\right)}W^{\left(u\right)}\right)$$

where ${\hat{A}}^{\left(u\right)}={\tilde{D}}^{{\left(u\right)}^{-1/2}}{\tilde{A}}^{\left(u\right)}{\tilde{D}}^{{\left(u\right)}^{-1/2}}$, $\tilde{A}=A+I_{N}$ means the adjacency matrix combining self‐connection, $\sigma \left(\cdot \right)$ represents a non‐linear function like ReLU or sigmoid, and $W^{\left(u\right)}$ is a weight matrix. Therefore, the new representation $H_{\text{drug}}$ of the drugs can be obtained through the drug similarity matrix $A^{\left(\text{drug}\right)}$ and the drug feature $X^{\left(\text{drug}\right)}$, and the new representation $H_{\text{protein}}$ of the protein can be obtained in the same way.

### 3.4 Reconstructing drug‐target matrix

According to the obtained drug and target characteristics, we need to reconstruct the drug‐target matrix for the purpose of prediction. Topology‐preserving learning of the node embedding^12^ is a proved good way to reconstruct the drug‐target prediction matrix. Given n drug nodes and m protein nodes, the reconstructed DTIs matrix can be expressed as:

(13) $$Y_{\mathrm{DT}I_{\text{reconstruct}}}=H_{\text{drug}}D_{r}P_{r}^{T}H_{\text{target}}^{T}$$

where $D_{r}\in R^{d\times n},P_{r}\in R^{d\times m}$ are specific mapping matrices of drug and protein, m and n represent the number of drugs and proteins, respectively, and r means a protein interaction.

The above equation states that the values of the edge mapping of the drug features and the target features through the mapping functions $D_{r}$ and $P_{r}$ can be reconstructed by doing the inner product of the mapped vectors. Natarajan and Dhillon et al.^28^ also used similar reconstruction strategies to solve the prediction problem. In the training process, the summation of the squared reconstruction errors of all edges is minimised by learning unknown parameters. So, given a drug‐target edge weight vector Y, we define the reconstruction loss of the edge weight value as:

(14) $$\min L={\left(Y-Y_{\text{reconstruct}}\right)}^{2}=\sum_{i}^{n}\sum_{j}^{n}{\left(y_{\mathrm{ij}}-h_{i}D_{\mathrm{ri}}P_{\mathrm{rj}}^{T}x_{j}^{T}\right)}^{2}$$

By minimising the final objective function, gradient descent training can be carried out.

### 3.5 Pseudocode of NGCN

The pseudocode for NGCN is provided in Algorithm 1 below.

ALGORITHM 1 **: Pseudocode of NGCN**

**Input**: Drug similarity matrixs, $A_{i},i\in \left[1,4\right]$; Protein similarity matrixs, $A_{j},j\in \left[5,7\right]$; **Output**: Reconstructed drug‐target matrix, $Y_{\mathrm{rec}}$;

1. Run random walk with restart on multi‐networks;
$S\leftarrow \mathrm{RWR}\left(A\right)$
2. Use diffusion component analysis (clusDCA) to perform feature reduction and feature extraction on the diffusion state set $R_{c_{1}}=\left\{S_{1},\ldots ,S_{4}\right\}$ of the drugs and the diffusion state set $R_{c_{2}}=\left\{S_{5},\ldots ,S_{7}\right\}$ of the proteins;
$X_{\text{drug}}\leftarrow \text{clusDCA}\left(R_{c_{1}}\right)$
$$X_{\text{target}}\leftarrow \text{clusDCA}\left(R_{c_{2}}\right)$$
3. Apply spectral graph‐based convolutional neural network to update the features of drugs and targets;
$H_{\text{drug}}\leftarrow \mathrm{Cov}\left(A_{\text{drug}},X_{\text{drug}}\right)$
$H_{\text{target}}\leftarrow \mathrm{Cov}\left(A_{\text{target}},X_{\text{target}}\right)$
4. Reconstruct the drug‐target matrix $Y_{\mathrm{rec}}$;

**return** $Y_{\mathrm{rec}}$;

- Step 1: the diffusion state $S_{i}$ for drug or target is derived by performing RWR algorithm (as shown in Equ 2) on each network.
- Step 2: clusDCA takes the diffusion state set $R_{c_{1}}=\left\{S_{1},\ldots ,S_{4}\right\}$ of the drug and the diffusion state set $R_{c_{2}}=\left\{S_{5},\ldots ,S_{7}\right\}$ of the protein as input to perform feature reduction for the node features, and obtain important topological feature information of nodes from the diffusion states.
- Step 3: the spectral graph‐based convolutional neural network is constructed according to Equ 11. Target features and the drug features mentioned above are updated.
- Step 4: the drug‐target matrix $Y_{\mathrm{rec}}$ is reconstructed by Equ 13, after obtaining the updated features $H_{\text{drug}}$ and $H_{\text{target}}$.

## 4 EXPERIMENTAL RESULTS

### 4.1 Dataset

In the whole training process, the dataset of our experiment is the same as that used by Luo et al.^27^ There are four types of nodes in the dataset including drug nodes, protein nodes, disease nodes and side effect nodes. There was no exception; those isolated nodes were excluded.

The dataset includes two kinds of similarity network and six types of association networks. The latter consists of drug‐protein association network,^30^ protein–protein association network,^31^ drug–drug interaction network,^30^ drug‐disease network^32^ and protein‐disease association network^32^ and drug‐side effect network.^33^ These networks can be used to construct corresponding similarity networks with respect to proteins and drugs. Among them, the former is generated by the similarity of the gene sequence of proteins, and the latter is constructed by the similarity of the medical chemical structure.

### 4.2 Superiority in DTI prediction

A drug‐target pair with a known interaction is considered a positive sample, and a drug‐target pair with an unknown interaction is generally viewed as a negative sample. To measure the performance of NGCN in predicting DTIs, we first performed 10‐fold cross‐validation on all positive pairs and a set of randomly sampled negative pairs, whose number was 10 times as many as that of positive samples. This scenario basically stimulated the practical situation in which the DTIs are sparsely labelled. For each fold, a randomly chosen subset of 90% positive and negative pairs was used as training data to construct the heterogeneous networks and then train the parameters of NGCN, and the remaining 10% positive and negative pairs were held out as the test set.

We compared NGCN with six baseline methods, including NeoDTI,^12^ DTINet,^27^ BLMNII,^34^ MOLIERE,^35^ NetLapRLS^36^ and HNM.^37^ Two evaluation indicators including AUPR (the area under the precision‐recall curve) and AUROC (the area under the receiver operating characteristic curve) were used to measure performance.

**FIGURE 2:**
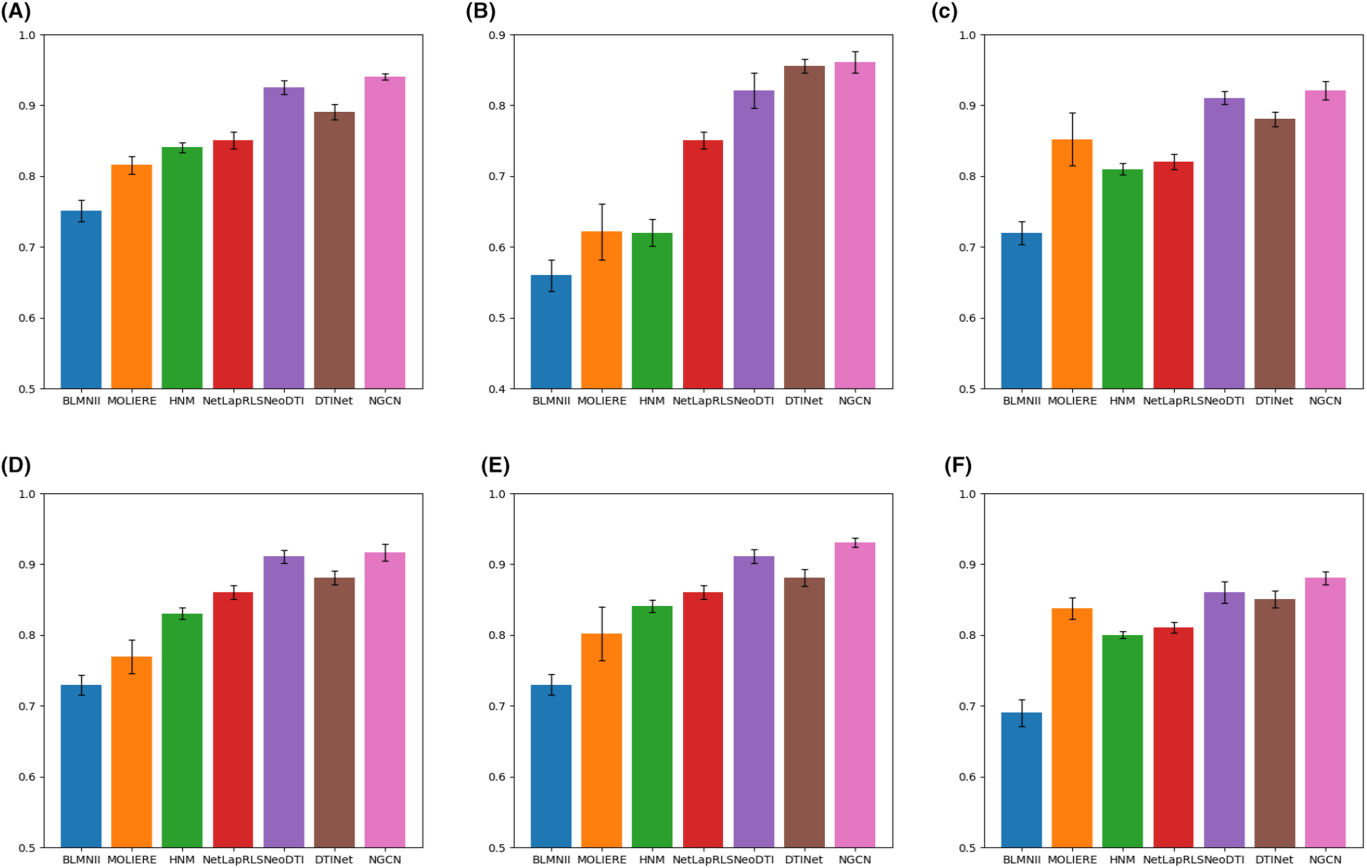
Comparison between *NGCN* and related methods. We apply 10‐fold cross‐validation in our experiments and compare *NGCN* with six other prediction methods (including *NeoDTI*, *DTINet*, *BLMNII*, *MOLIERE*, *NetLapRLS* and *HNM*) in terms of prediction effects. The y‐axis describes *AUPRC* for measuring prediction performance. (A) Specifying proportion 1:1 for positive and negative examples. (B) Specifying proportion 1:10 for positive and negative examples. (C–F) Several strategies to remove data redundancy: (C) Removing DTIs sharing similar drugs. (D) Deleting DTIs sharing similar diseases. (E) Deleting DTIs with drugs showing similar side effects. (F) Pruning DTIs with similar drugs or proteins.

In Figure 2, we can observe that NGCN has better performance than other methods, which is higher than the best method. In addition to known DTI data, the chemical structure, protein sequence information and other properties of drugs and targets can also be determined through their various functional roles in biological systems, such as protein–protein interactions and drug‐disease associations. By integrating disparate information from heterogeneous data sources, methods such as DTINet, NeoDTI and HNM can further improve the accuracy of DTI predictions. However, there are still some limitations to these approaches that need to be addressed. For example, HNM method only considers three different types of data to make relationship prediction, thus discarding a lot of valuable information. In addition, methods such as BLMNII and MOLIERE only take relatively simple forms (such as bilinear linear or log‐linear functions), which may not be sufficient to capture complex hidden features behind heterogeneous data. The reason for NGCN's excellent performance lies in its initial utilization of RWR to compute the diffusion state of nodes for each network, followed by its integration with clusDCA for dimensionality reduction operations. In this manner, the noise in the data is substantially reduced. The spectral graph convolutional neural network is then employed to further learn drug or target features. Unlike DTINet, where predictions are solely based on dimensionless diffusion states, NGCN enhances its predictive capability by optimizing features using the graph convolution model, thereby achieving superior results.

To verify the performance of NGCN under sparse positive samples, we changed the number of samples and specified the proportion 1:10 for positive and negative examples. It is observed that the performance of all other algorithms decreased. In contrast, NGCN still achieved the best prediction performance. This shows that even in the case of sparse labelling, the prediction performance of other methods is still inferior to the NGCN method. In addition, we performed statistical significance tests at the 95% confidence level on the results of the NGCN and NeoDTI (the best performance method in the comparison experiment) using 10‐fold cross‐validation. The results show that the observed differences between the two methods are statistically significant.

Since the data may be redundant, for example, there are multiple homologous proteins for one protein or multiple highly similar drugs for one drug in the dataset, which may negatively affect the performance. Therefore, we applied the same strategy as Luo et al. to reduce the impact of data redundancy by removing drug‐target associations of similar drugs or targets in the drug‐target interaction matrix. We eliminated drug‐target associations in which the Jaccard similarity in the association network was greater than 0.6, the structure similarity score in a medicinal chemical similarity network exceeds 0.6, and the identity score in the protein–protein sequence similarity network exceeds 0.4.

In the experiment, we kept the ratio 1:1 for negative and positive samples. As expected, after the deletion of similarity, NGCN performance declined but was still superior to other baseline methods.

### 4.3 Effects of NGCN components

In this paper, we propose a multi‐network integration algorithm, termed as NGCN and apply it on drug‐target interactions prediction using GCN model. We use GCN to aggregate neighbourhood features to further improve the availability of features. The spectral‐based graph convolution network (GCN) method introduces filters from the perspective of graph signal processing to define graph convolution, where the graph convolution operation is interpreted as removing noise from the graph information. In order to evaluate the performance of GCN part, we implemented a multi‐networks integration framework without updating features (i.e. use the spectral‐based graph convolutional neural network for updating features), to evaluate the effects of the proposed NGCN. We compared our method, NGCN, with these various approaches to validate the effects of the feature updating operation, and the experimental results are reported in Table 1. The results show that the feature updating operation of our proposed NGCN algorithm demonstrates substantial superiority on the task of predicting drug‐target interactions.

**TABLE 1:** Performance of drug‐target interaction prediction under different settings (No. positive:No. negative = 1:1).

Feature‐update	Drug‐dimension	Protein‐dimension	AUPR	AUROC
NO	100	200	0.889	0.863
YES	100	200	0.901	0.880
NO	200	200	0.894	0.875
YES	200	200	0.914	0.895
NO	100	400	0.924	0.904
YES	100	400	0.926	0.901
NO	200	400	0.921	0.900
YES	200	400	**0.943**	**0.910**

*Note*: The best performance results are highlighted in bold.

### 4.4 Robustness

In the experiment, we mainly evaluated the influence of parameters and the robustness of NGCN. The robustness of NGCN was tested by changing the number of networks related to the drugs or target, the feature dimension and the hyperparameters of NGCN. All experimental results were obtained by adopting 10‐fold cross‐validation.

**FIGURE 3:**
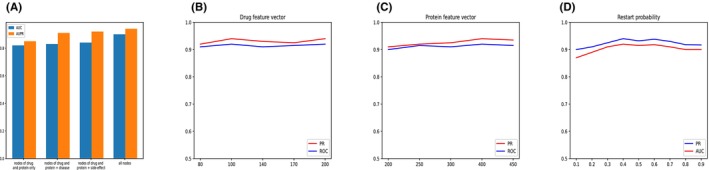
Robustness of NGCN. (A) Effects of aggregating multiple heterogeneous networks. (B) Effects of drug dimensions. (C) Effects of protein dimensions. (D) Effects of restart probability.

We start from examining the effects from aggregating multiple heterogeneous networks on the predicted results. We only used drug‐protein association matrices (i.e. drug similarity network, drug–drug association network, protein–protein association networks, protein similarity network and drug‐protein association network) to conduct performance evaluation. Through training, we observed that the prediction performance was significantly reduced compared to the original model, NGCN, which obtained the features from all networks. We also increased the number of networks associated with disease and side‐effects. Under expectation, it is observed that the prediction performance could be improved by adding drug‐ and protein‐related networks. Experiments show that aggregating heterogeneous information in the networks generated by multiple data sources is able to improve the prediction accuracy. Furthermore, we applied NGCN to predict drug‐target interactions under different feature dimension conditions and compared the AUPR values of the predicted results. According to the experiment of Wang et al.,^29^ the dimension of the feature vector in the diffusion state dimension of 10%–20% achieved the best results. We expanded the scope of the study to 10% to 30%, and we set the drug dimension to 80, 110, 140, 170, 200 and protein dimension to 200, 250, 300, 350 and 400. From the observations, there was little impact on the predicted results (see Figure 3).

We further investigated the impact of hyperparameters on experimental performance. Here, we mainly studied the influence of restart random walk probability *p* on the experimental results. In the test, we considered the restart probability value between 0.4 and 0.7 to observe the performance stability under different probabilities. In Figure 3, it can be seen that when the restart probability is varied from 0.4 to 0.7, NGCN achieves stable performance. Thus, these parameters have little impact on the experimental performance.

To validate the robustness and scalability of our proposed approach, we evaluated it on four drug‐target interaction datasets created by Yamanishi et al.^6^: Enzyme, Ion Channels, G‐protein‐coupled receptors (GPCR) and Nuclear receptors. The datasets are available at http://web.kuicr.kyoto‐u.ac.jp/supp/yoshi/drugtarget/. We compared NGCN with six other prediction methods in terms of prediction effects using 10‐fold cross‐validation. Table 2 shows that NGCN outperforms other methods, indicating that our approach can be applied to other drug‐target interaction prediction scenarios.

**TABLE 2:** The AUC values of six existing methods and our proposed method on the four datasets using 10‐fold cross‐validation.

Dataset	*NetLapRLS*	*BLMNII*	*HNM*	*DTINet*	*MOLIERE*	*NeoDTI*	*NGCN*
Enzyme	0.945	0.951	0.949	0.953	0.961	0.957	**0.973**
Ion channels	0.953	0.957	0.924	0.971	0.963	0.961	**0.975**
GPCR	0.928	0.939	0.940	0.937	0.944	0.945	**0.957**
Nuclear receptors	0.891	0.914	0.894	0.921	0.913	0.938	**0.944**

*Note*: The best performance results are highlighted in bold.

### 4.5 Identification of new targets for known drugs

We analysed the predicted scores of DTIs in the results. In the unknown DTIs prediction, we selected the top 10 predicted scores of DTIs, and three of these DTIs can be supported by previous studies in the literature. For example, *nifedipine* is a drug that has been approved to suppress spontaneous arrhythmia, and our NGCN predicted that SCN5A, which interacts with *nifedipine*, plays an important role in ventricular arrhythmia.^38^, ^39^ COX‐2 encoded by the PTGS2 gene is an inducible enzyme that can be highly induced by pro‐inflammatory cytokines and tumour promoters in various cells. And *nifedipine* inhibits the expression of COX‐2 of human OA chondrocytes.^40^ This interaction was also predicted by NGCN. In addition, *nifedipine* has a good clinical effect on high‐altitude pulmonary oedema and has been approved for adjunctive treatment.^41^ *Nifedipine* was predicted by NGCN to interact with NR3C1, and NR3C1 gene polymorphisms are associated with high‐altitude pulmonary edema.^42^ In general, the new DTIs predicted by NGCN are supported by literature, which further demonstrates the powerful predictive ability of our model.

## 5 CONCLUSIONS

The challenge of integrating information from multiple networks for DTI prediction mainly arises from the complexity and heterogeneity of different drug‐related networks, as well as from the high‐dimensional, incomplete and noisy nature of data. To solve this problem, we propose a novel method called NGCN, which updates features through GCN by fusing features from multiple networks. NGCN analyses the structural characteristics of each network through a network diffusion process and extracts low‐dimensional hidden vectors of the network. It has demonstrated significant improvement over baseline approaches for DTI prediction by leveraging updated features via convolutional optimization. Moreover, NGCN is an extensible framework that can incorporate more information about drugs and targets, offering flexibility to enhance features and integrate more heterogeneous information to improve the prediction accuracy. In our future work, we will focus on two main aspects to enhance our approach. Firstly, we will enhance the utilisation of biological information by integrating more diverse network data into our framework, leading to a more comprehensive understanding of drug‐target interactions. Secondly, we will address the issue of significant differences in node degrees within the graph network to ensure effective extraction of information from low‐degree nodes. These enhancements aim to achieve more precise and reliable prediction of drug‐target interactions.

## AUTHOR CONTRIBUTIONS

**Junyue Cao:** Conceptualization (equal); formal analysis (equal); funding acquisition (equal); investigation (equal); methodology (equal); project administration (equal); software (equal); writing – original draft (equal); writing – review and editing (equal). **Qingfeng Chen:** Conceptualization (equal); funding acquisition (equal); project administration (equal); supervision (equal). **Junlai Qiu:** Software (equal); validation (equal); writing – review and editing (equal). **Yiming Wang:** Software (equal); validation (equal). **Wei Lan:** Validation (equal); writing – review and editing (equal). **Xiaojing Du:** Visualization (equal); writing – review and editing (equal). **Kai Tan:** Validation (equal).

## CONFLICT OF INTEREST STATEMENT

The authors declare that they have no known competing financial interests or personal relationships that could have appeared to influence the work reported in this paper.

## Data Availability

The source code and data can be available at https://github.com/Junyue28/NGCN/.
